# Meteorological and landscape influences on pollen beetle immigration into oilseed rape crops

**DOI:** 10.1016/j.agee.2017.03.008

**Published:** 2017-04-01

**Authors:** Matthew P. Skellern, Sue J. Welham, Nigel P. Watts, Samantha M. Cook

**Affiliations:** a Rothamsted Research, Harpenden, Hertfordshire, AL5 2JQ, UK; b VSN International, 2 Amberside House,Wood Lane, Hemel Hempstead, HP2 4TP, UK; c Current address: Stats4Biol, Welwyn Garden City, UK

**Keywords:** *Brassicogethes*, Decision support systems, Insecticide resistance, Integrated pest management, *Meligethes aeneus*, Monitoring trap, Sustainable intensification

## Abstract

Heavy reliance on pesticide inputs to maintain crop yields has been an important aspect of agricultural intensification. Insecticide use has had detrimental impacts on pollinators and natural pest control agents, contributing to a decline in associated ecosystem services, and has also led to resistance development in pest populations. Throughout Europe, in oilseed rape (*Brassica napus* L.) crops, prophylactic use of insecticides against pollen beetles (*Meligethes aeneus* F. also known as *Brassicogethes aeneus*) has led to such issues, and there is an urgent need to develop more sustainable pest management practices for the crop. Although advice is available to oilseed rape growers regarding control thresholds, it may not be adhered to due to the expense of pollen beetle monitoring relative to the inexpensive cost of pyrethroid insecticides. Thus, the key to reducing prophylactic insecticide applications may lie with improved, less labour intensive methods of pollen beetle monitoring. For these to be realized, a better understanding is needed of the effects of agri-landscape features and meteorological conditions on pollen beetle immigration into the crop. In this study, based on data from four years of pollen beetle monitoring on a total of 41 field sites, we model the effects of meteorological (wind speed and direction, rainfall and accumulated temperature) and landscape (areas of woodland, residential gardens, the current and previous seasons’ oilseed rape crops, and lengths of hedgerows and treelines) variables on directional sticky trap catches, at both the single trap and field scales. Meteorological variables, particularly accumulated temperature and wind speed were more important than landscape variables in predicting the abundance of pollen beetles immigrating into OSR fields. Sticky traps that were facing downwind caught more beetles than those that were facing across-wind or upwind; this is the first study to show at a landscape-scale, direct evidence for use of upwind anemotaxis by pollen beetles at the point of entry during immigration into the crop. At the field scale, the area of oilseed rape grown in the previous season was found to be positively related to trap catch, but no relationships with other landscape variables were found. Optimally-placed monitoring traps could complement existing decision support systems to reduce pollen beetle monitoring effort and encourage use of insecticides only when control thresholds are breached, thus enhancing the sustainability of oilseed rape production. Knowledge of the area of oilseed rape crops grown during the previous season in the surrounding landscape could contribute to risk assessment of potential pest pressure for individual OSR crops.

## Highlights

• Pollen beetle immigration into oilseed rape (OSR) was influenced by weather and landscape variables.
• Accumulated temperature and wind speed were most important for immigration.
• Beetles flew upwind into OSR crops; downwind-facing trap catches were greatest.
• Local OSR area in the previous season was positively related to field-scale trap catch.
• These findings could help minimise beetle monitoring effort and reduce prophylactic spraying.

## 1 Introduction

The heavy reliance on pesticide inputs associated with post-WW2 agricultural intensification, along with habitat loss and a reduction in cropping diversity, has contributed to environmental degradation and declining biodiversity within farmed landscapes (Stoate et al., 2001, Robinson and Sutherland, 2002). In particular, the impact of pesticides on pollinators and natural pest control agents has played a role in the deterioration of associated ecosystem services which are vital for sustainable food production (Goulson, 2013, Vanbergen, 2013, Roubos et al., 2014). Furthermore, sustained use or overuse of insecticides has frequently led to development of resistance (Heckel, 2012), threatening the sustainability of existing pest management practices and potentially impacting agricultural productivity. As a result of these issues, and due to increasing public concern, there is an urgent need to reduce pesticide use and develop pest management practices that are more ecologically sustainable.

Throughout Europe, pollen beetles (*Meligethes aeneus* F.; also known as *Brassicogethes aeneus*) are a major pest of oilseed rape (OSR) (*Brassica napus* L.) (Williams, 2010), a crop that has seen significant production increases over recent years (from 19.0 M tonnes produced within the EU in 2008 to 21.7 M tonnes in 2015; Eurostat, 2016a), mostly due to rising demand for biofuel use (Eurostat, 2016b). The beetles overwinter as adults in the leaf litter of hedgerows, woodlands and grassy areas before emerging in early spring (Nilsson, 1988, Rusch et al., 2012a), when they feed on pollen from a range of spring flowers before seeking brassicaceous plants for oviposition (Free and Williams, 1978, Ouvrard et al., 2016). The beetles use both visual and olfactory cues for host plant location (Williams and Cook, 2010, Cook et al., 2007, Cook et al., 2013) and have been observed to use upwind anemotaxis to fly towards their host crops (Evans and Allen-Williams, 1994, Williams et al., 2007, Moser et al., 2009). They usually arrive in OSR crops at or around the green bud stage (BBCH growth stage code 51; Lancashire et al., 1991). Feeding damage caused by adults results in yield loss from bud abscission (Nilsson, 1987, Alford et al., 2003). The beetle larvae feed within the buds and flowers for around two weeks before dropping to the ground to pupate in the soil. New generation adults emerge during summer and are again polyphagous on several families of flowering plants before overwintering (Alford et al., 2003, Williams, 2010).

Although pollen beetle-related yield losses can approach 70% in spring crops (Nilsson, 1987), winter crops have often developed beyond the susceptible bud stages by the time the beetles arrive (Williams, 2010), and control thresholds are breached infrequently (Ellis and Berry, 2012). Despite this, pollen beetles in winter OSR are frequently the target of spring insecticide treatments, usually pyrethroids (Williams, 2010, Garthwaite et al., 2011), many of which are applied as prophylactic ‘insurance’ sprays (Thieme et al., 2010). This overuse has damaging effects on beneficial insects (Mänd et al., 2010, Hanson et al., 2015) and has led to increasingly widespread pyrethroid resistance, threatening to limit the arsenal of products remaining active against the pest (Thieme et al., 2010, Zimmer and Nauen, 2011, Nauen et al., 2012, Heimbach and Mueller, 2013).

The use of prophylactic treatments for pollen beetle control has been encouraged by the low cost of pyrethroid insecticide products, and application costs are often also minimal as treatments are frequently applied as sprayer tank mixes, at the same time as fungicide programs (Thieme et al., 2010). Although advice on pollen beetle monitoring and control thresholds is available to farmers (e.g. AHDB-HGCA, 2013), its uptake may be discouraged by costly labour intensive monitoring requirements and poor reliability due to variation in spatial distributions of the pest, which are often patchy (Cook et al., 2004, Ferguson et al., 2003, Gotlin Culjak et al., 2016). Thus, the key to reducing prophylactic insecticide applications in OSR and improving the sustainability of the crop may lie with improved, less labour intensive methods of pollen beetle monitoring. The recent development of on-line weather-based decision support systems (DSSs) such as proPlant expert (Johnen and von Richthofen, 2013) represents progress towards this goal, by providing regional forecasts of migration risks which reduce monitoring requirements (Ferguson et al., 2016). However, as landscape factors such as woodlands (as overwintering sites) and oilseed crops in the previous and current seasons (as potential sources and alternative sinks, respectively) are known to influence local beetle abundance and crop damage (e.g. Valantin-Morison et al., 2007, Zaller et al., 2008b, Rusch et al., 2012b, Rusch et al., 2013), the development of models based on both meteorological and landscape factors could be used to further refine such DSSs, and also to help determine optimal placement of traps for monitoring immigration into the crop.

In the current study, based on data from four years of pollen beetle monitoring on a total of 41 field sites, we model meteorological and landscape influences on pollen beetle immigration into the crop as measured by directional sticky trap catches, at both the single trap and field scales. We hypothesize that (i) temperature, rainfall and wind speed will affect trap catches by determining when conditions are optimal for beetle immigration into the crop, (ii) since the beetles may be expected to fly towards the crop using upwind anemotaxis, wind direction will affect the direction from which they enter, and (iii) that landscape features will interact with wind direction to affect trap catches by influencing beetle abundance at source.

## 2 Materials and methods

### 2.1 Field sites and sticky trapping

As part of a project to develop an integrated pest management strategy for pollen beetles (Cook et al., 2014), beetle immigration into a total of 178 winter OSR fields, distributed throughout the main UK arable cropping regions, was monitored by sticky trapping during the March–early May periods of 2008–2011. For the purposes of this study, data were analysed from a subset of 41 sites (4, 7, 18 and 12 sites from 2008, 2009, 2010 and 2011, respectively); (see Fig. S1 and Table S1 in Supplementary material for further details). With the exception of sites at Rothamsted (Hertfordshire) and Woburn (Bedfordshire), where trapping was conducted by Rothamsted staff, trapping at all other sites was done by volunteer farmers or crop consultants. Some of the volunteers ran traps on more than one field of a farm, either in the same or different years. Sites were selected for analysis based on several criteria. The volunteers were required to have followed experimental protocols accurately, and provided good information on the locations of oilseed rape crops within a 1000 m radius of the traps for the year of sampling and the previous year. In order to be sure that the immigration period had been identified correctly, the number of beetles trapped per day was required to show a peak followed by a decline; sites with no clear peak were excluded.

**Fig. 1:**
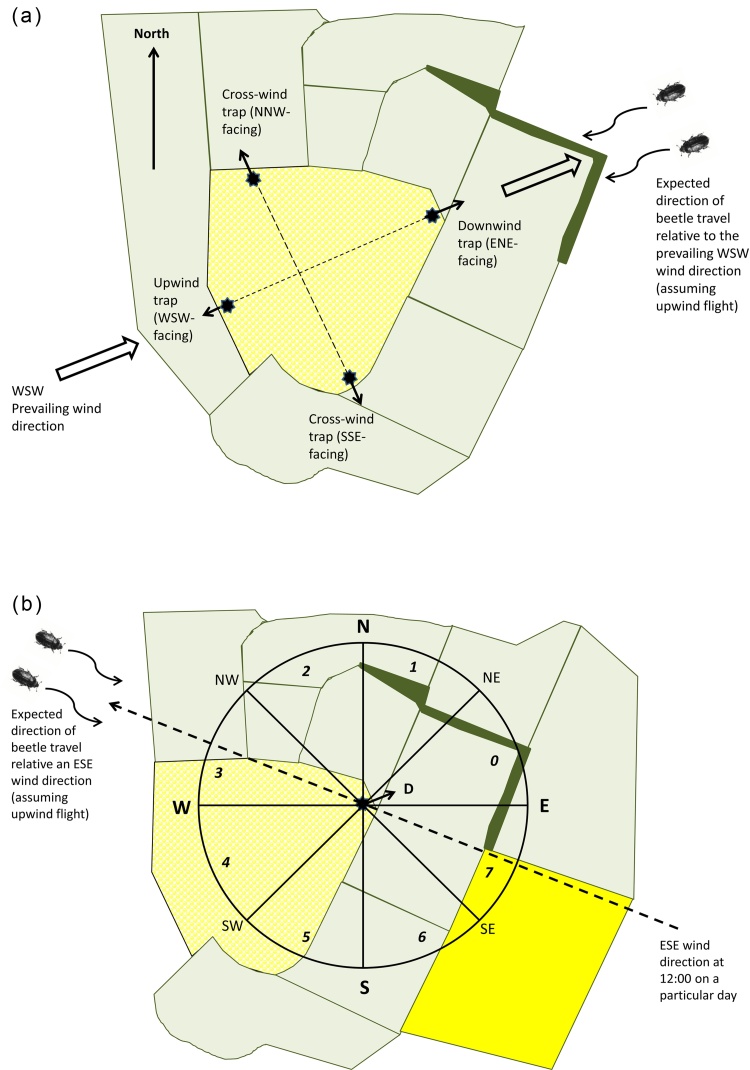
(a) Typical site layout showing directional sticky trap placement in oilseed rape crops relative to an assumed WSW prevailing wind direction (hollow arrows) and the boundaries of a sampled field (textured shading). Upwind (WSW-facing) and downwind (ENE-facing) traps (black stars with arrows to indicate facing direction) were placed 3 m into the crop on opposite sides of the field, along the plane of the WSW-to-ENE prevailing wind direction (short-dashed line). Fifteen of the sites had additional NNW- and SSE-facing cross-wind traps, placed on a NNW–SSE plane (long-dashed line), perpendicular to that of the prevailing wind. When the wind is from the prevailing WSW direction, pollen beetles flying upwind are expected to enter the crop from the ENE. (b) Landscape mapping zones and assignment of wind and trap directions to specific directional segments or ‘octants’. The circular area represents the 1000 m-radius zone within which landscape features were mapped in relation to a downwind trap (D; ENE-facing), and is divided into directional octants (labelled *0*–*7*). Traps were assigned to the octant corresponding to the direction that they were facing (in the case of the downwind trap shown, octant *0*). If the wind direction was from the ESE at 12:00 on a particular day (dashed straight arrow), ingress of upwind-flying beetles would be expected from a WNW (downwind) direction, and hence wind direction in this situation would be assigned to octant *3*. The difference between wind direction and trap direction can then be calculated in terms of number of octants (in this case there was a 3-octant difference). However, if the wind direction was from the WSW, as in (a), then both the downwind trap and wind direction would be assigned to octant *0* (trap and wind direction aligned, a difference of 0 octants). Note that for modelling at the individual trap scale, only landscape features from the octant facing the trap and its two immediate neighbours (in this case octants *0*, *1* and 7) were used.

At each site, either two or four yellow sticky traps (standard ‘wetstick’, 10 cm x 20 cm) (Oecos, Kimpton, Hertfordshire, UK) were placed on different sides of the field. At all sites, one trap was placed upwind and another downwind along the plane of an assumed WSW prevailing wind direction, and on 15 of the sites, two additional traps were placed in ‘cross-wind’ positions, at right angles to this plane (Fig. 1a). The sticky traps were clipped onto a plastic mount and placed on top of an extendable metal pole so that they could be maintained just above crop canopy height. The traps were angled at 45° to the vertical as this orientation has been shown to be effective for trapping pollen beetles (Blight and Smart, 1999). Traps were placed 3 m into the crop from the field edge and were orientated to face outwards, in order to capture incoming beetles.

Trapping commenced on or shortly after 1st March each year and was continued until the crop reached early flowering (BBCH growth stage code 61, Lancashire et al., 1991). Where possible, traps were changed every 3–4 days, but in practice intervals depended on volunteer time availability, and were often longer (full range 2–21 days but with c. 80% of intervals < 7 days). The collected traps were covered with a polyethylene sheet and returned to Rothamsted for the pollen beetles to be counted. For each site, the sample representing peak beetle count was identified (from the total count from upwind and downwind traps only), and all samples up to and including that date were used for analyses, as representing the period of immigration into the crop. A total of 309 sticky trap samples were used in the analyses.

### 2.2 Landscape data

Landscape features were digitally mapped within a 1000 m-radius around each trap using Google Earth (Google Inc., Mountain View, California, USA). The 1000 m scale was chosen as it lies within the range of scales over which previous studies had shown an influence of landscape features on pollen beetle abundance or herbivory (e.g. Thies et al., 2003, Zaller et al., 2008b, Moser et al., 2009). The features mapped were included on the basis of their potential to provide overwintering habitats for the beetles (woodlands) or act as potential barriers to their movement (hedgerows and lines of trees), or because of their importance as sites for feeding and reproduction (OSR crops − both in the year of trapping and previous year, and residential gardens). Permanent features not already shown on Ordnance Survey base maps (accessed via the UK Digimap service; http://digimap.edina.ac.uk) were identified from Google Earth satellite imagery, while information on the location of OSR crops in the vicinity was provided by the data contributors. ArcGIS 9.2 (ESRI, Redlands, California, USA) was used to extract areas of landscape features (or lengths of linear hedgerows and treelines) from within each of eight 45° sectors, or ‘octants’, that were created to subdivide the 1000 m-radius circular mapped zone into directional components (Fig. 1b). As few fields in our study were particularly large, there was some overlap between landscape features present in sectors around upwind and downwind traps. However, the landscape variables were used as explanatory variables in our model and as the method does not require statistical independence within the explanatory variables, this was not considered a problem from a statistical point of view. A summary of the landscape data is given in Table S2.

### 2.3 Meteorological data

Meteorological data were obtained from the nearest UK Meteorological Office recording station to each field site, with the exception of Rothamsted and Woburn, where local on-farm data were available (see Table S1 for details of the meteorological stations and their approximate distances from the trap sites.) Daily summaries of the minimum and maximum temperature (°C), rainfall (mm), and wind speed (m/s) and direction were obtained for the duration of sampling at each site.

As maximum temperature is unlikely to be a reliable guide to the temperature over the day, an accumulated temperature measure was calculated. This used a saw-tooth approximation which assumed that the minimum and maximum temperatures occurred at 05:00 and 15:00 respectively, and used linear interpolation between these points. No beetles were found on traps when the maximum daily temperature was less than 10 °C, and so 10 °C was used as the baseline temperature. The accumulated temperature (degree-half-days; dhd) was calculated as the integral of the saw-tooth function during the period 06:00–18:00 (assumed daylight hours) when the interpolated temperature exceeded the baseline 10 °C (Fig. S2). For days when the temperature did not reach 10 °C, the accumulated temperature was assigned as zero.

Although wind speed and direction data were available on four occasions throughout the day, (at 00:00, 06:00, 12:00 and 18:00), the reading at 12:00 was chosen as being most relevant to beetle activity as it occurred during the warmer part of the day most likely to coincide with immigration. For each day that a trap was running, wind direction at 12:00 was assigned to one of the octants used for landscape data. This octant corresponded to the expected direction of beetle ingress into the crop if beetles flew upwind, and therefore represented the direction that the wind was going *to*, (i.e. the ‘downwind direction’), as opposed to the direction that it was coming *from* (i.e. the ‘upwind direction’ used in the standard definition of wind direction). For example, if the wind direction was from ESE then it was assigned to octant 3 (the WNW-facing octant opposite; Fig. 1b). Each trap was assigned to the octant corresponding to the direction it was facing (out of the crop). For example, a downwind trap facing ENE would be assigned to octant 0. The difference between the actual downwind direction (variable) and the direction of the trap (fixed) was calculated in terms of number of octants (Fig. 1b), taking the shortest route around the circle. For example, a trap facing ENE (octant 0) would have a deviation of 3 octants for either wind direction ESE (octant 3) or NNE (octant 5). A trap facing ENE (octant 0) with wind direction WSW (also octant 0) would have a deviation of 0 octants (trap and wind direction aligned).

Summaries of the weather variables are given in Figs. S3 (temperature), S4 (wind speed and direction), and S5 (rainfall).

### 2.4 Modelling

All analyses and modelling were done using GenStat (17th edition, VSN International, Hemel Hempstead, UK). Before building a model in terms of the explanatory variables, a simple exploratory model was used to ascertain the different sizes and sources of variability and the distribution of the data (details in Section S1 of Supplementary material). The residuals from this analysis and the mean squared residuals were inspected for indications of variance heterogeneity, in particular for any pattern of variance increasing as a quadratic function of the mean, which would be more consistent with a negative binomial distribution than with a Poisson distribution (ver Hoef and Boveng, 2007). The negative binomial distribution was parameterised with mean μ, aggregation parameter k and a variance function of the form:

$$V(\mu )=\mu \left(1+\frac{\mu }{k}\right)$$

#### 2.4.1 Modelling weather and landscape variables

To explore the effect of weather and landscape on trap catches, a generalised linear mixed model (GLMM) with a composite link function and a negative binomial distribution was used. Since there was a discrepancy between the scale of the weather data (daily) and the sample period for each trap (2–21 days; see Section 2.1), the modelling process required a mechanism to model daily beetle counts as a function of daily weather and then accumulate these counts over the sample period to predict total trap count; this was achieved via the composite link function. As the weather and landscape variables apply to days within fields and traps within fields, respectively, random terms corresponding to fields, days within fields and traps within fields were required to avoid pseudo-replication and ensure that fixed terms were tested at the correct level within the data structure; this was achieved by use of a GLMM. The composite link method of Thompson and Baker (1981) was implemented within the framework of a GLMM with penalised quasi-likelihood (PQL) estimation (Breslow and Claydon, 1993). The composite link allows accumulation of counts over the sampling period. Its implementation in the GLMM framework models allows the inclusion of random terms to reflect the structure of the data with an appropriate distribution for the observations. The presence of explanatory terms in the model is examined using *t*-tests. The model can operate at the scale of the field (accumulating over traps) or the individual trap. Explanatory variables were classed as weather (accumulated temperature, daytime rainfall, wind speed) or landscape (area of woodlands, gardens, OSR crops in the current and previous year, and lengths of treelines and hedges). In addition, an explanatory variable was included in all models to adjust for higher counts from fields on the Rothamsted farm, identified from the initial exploratory model (see Section S1 of the Supplementary material).

#### 2.4.2 Modelling at the field scale

The total count from the upwind and downwind traps in each field was used as the response when modelling at the field scale. Landscape variables were averaged over the upwind and downwind traps, and field was included as the only random term. The three meteorological variables were added to the model first, as linear and quadratic functions. Non-significant terms were then dropped from this interim model using backwards selection. The landscape variables were then each added individually into the model. Any landscape variables found to be significant were then added as a group to the model containing meteorological variables, and backwards selection repeated to drop any non-significant terms. Predictions for each variable selected were made at the scale of the daily total field trap catch, using fixed specified values of other variables. Confidence intervals for predictions were formed on the log-scale and then back-transformed.

#### 2.4.3 Modelling at the trap scale

Beetle counts from individual traps (upwind, downwind and cross-wind) from each field were used as the response when modelling at the trap scale. Relative wind direction (as defined in Section 2.3 and Fig. 1b) was added to the set of explanatory variables used for modelling at the field scale. Based on the assumption that the beetles take a reasonably direct route to the field, landscape data from only the three octants facing each trap (i.e. the octant assigned to the trap plus one either side; Fig. 1b) were used to calculate the landscape explanatory variables. Field, day within field and trap within field were included as random terms in the model.

The modelling procedure was the same as that used for field-scale modelling (section 2.4.2), but included the additional meteorological variable, relative wind direction, parameterised as a factor with 5 levels (direction deviation of 0, 1, 2, 3, or 4 octants).

## 3 Results

The exploratory model indicated that a negative binomial distribution with aggregation parameter k = 5 gave acceptable residuals (Section S1 of Supplementary material) so this distribution was adopted for the models.

### 3.1 Modelling at the field scale

The modelling procedure identified the presence of a field site on the Rothamsted estate, wind speed, accumulated temperature, and the area of OSR crops in the previous year as significant explanatory terms for total field trap counts (Table 1). Details of the fitted model are given in Section S2.1 of the Supplementary material. The estimated values of fixed effects in the final model are shown in Table 1.

**Table 1:** Estimated parameters from the field-scale model. The t-ratio is calculated as parameter estimate/SE. F probability is the observed significance level from an approximate F-test on sequentially adding each term into the fixed model.

Term	Parameter	Estimate	SE	t-ratio	F prob
Constant (1)	α	0.6967	0.4819	1.445	0.154
Presence on Rothamsted estate (0/1) (*R*)	β_1_	2.1920	0.3364	6.516	<0.001
Linear wind speed (*w*, m/s)	β_2_	−0.4803	0.0404	−11.885	<0.001
Linear accumulated temperature (*a*, dhd)	β_3_	3.0094	0.3098	9.715	<0.001
Quadratic accumulated temperature (*a*^2^)	β_4_	−0.3277	0.0727	−4.510	<0.001
Linear area of OSR crops in previous year (*p*, ha)	β_5_	0.013789	0.006957	1.982	0.049

**Fig. 2:**
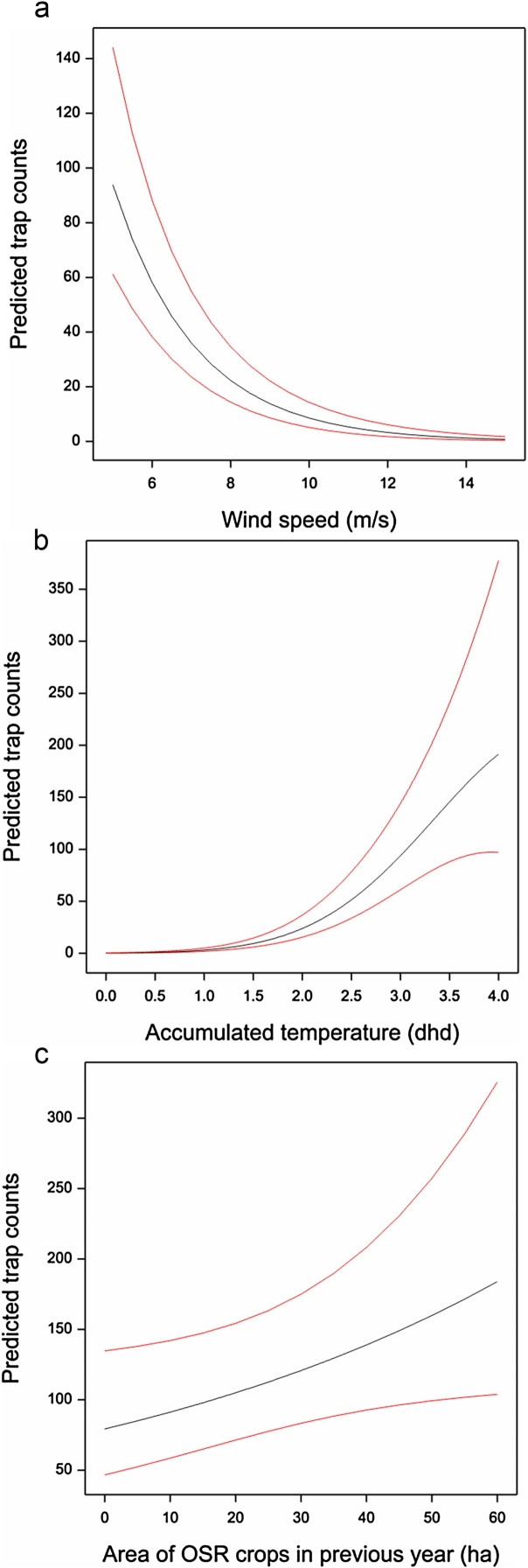
Predicted daily field catch of pollen beetles in traps placed in oilseed rape (OSR) crops (total of upwind and downwind traps) with 95% confidence limits for (a) wind speed (m/s) with 3 dhd accumulated temperature and 12 ha OSR in landscape in previous year, (b) accumulated temperature (dhd) with wind speed of 5 m/s and 12 ha OSR in landscape in previous year, (c) the area of OSR crops within a 1000 m radius with wind speed of 5 m/s and 3 dhd accumulated temperature.

There was a positive effect on daily total field catches of being located on the Rothamsted estate, giving a predicted ratio of beetle numbers at Rothamsted relative to non-Rothamsted fields of 8.95, ie. 8.95 times more beetles at Rothamsted sites if all other variables were equal, with 95% confidence interval (4.54, 17.67). Weather variables were more important (i.e. with larger t-ratios) as explanatory variables than landscape features (Table 1). Wind speed (m/s) showed a statistically significant negative linear relationship with log daily field catch (Table 1) shown in Fig. 2a with 95% confidence limits; field catches are expected to increase greatly at lower wind speeds. Accumulated temperature (dhd) showed a statistically significant positive linear relationship with log daily field catch, with a negative quadratic component (Table 1) shown in Fig. 2b. Field catches are expected to increase as accumulated temperature increases up to about 4.5 dhd, at which point they are predicted to plateau and then decline. Note that 4.5 dhd corresponds to a constant daytime temperature of 14.5 °C, and is in the top 0.5% of the observed sample so these predictions are based on little data. Rainfall was not a statistically significant variable and the only statistically significant landscape variable (using a P = 0.05 threshold) was a positive linear relationship on the log scale with area of OSR grown in the previous year (Fig. 2c).

### 3.2 Modelling at the trap scale

The modelling procedure identified the presence of a field site on the Rothamsted estate, wind speed, accumulated temperature, a deviation of 2–4 octants between downwind direction and trap direction, and the area of OSR crops in the previous year within the 3 octants facing the trap as important explanatory terms for trap counts (Table 2). Details of the fitted model are given in Section S2.2 of the Supplementary material. Estimates of the fixed effects from the final model are shown in Table 2.

**Table 2:** Estimated parameters from the trap-scale model. The t-ratio is calculated as parameter estimate/SE. The F probability is the observed significance level from an approximate F-test on sequentially adding each term into the fixed model.

Term	Parameter	Estimate	SE	t ratio	F prob
Constant (1)	α	−3.843	0.4234	−1.054	0.293
Presence on Rothamsted estate (0/1) (*R*)	β_1_	2.099	0.3210	6.540	<0.001
Linear wind speed (*w*, m/s)	β_2_	−0.531	0.0403	−13.174	<0.001
Linear accumulated temperature (*a*, dhd)	β_3_	3.795	0.3233	11.737	<0.001
Quadratic accumulated temperature (*a*^2^)	β_4_	−0.548	0.0857	−6.396	<0.001
Linear area of OSR crops in previous year (*p*, ha)	β_5_	−0.01903	0.007893	−2.411	0.018
Deviation of 1 octant in wind direction (*D* = 1)	δ_1_	0.019	0.1568	0.123	0.902
Deviation of 2 octants in wind direction (*D* = 2)	δ_2_	−0.387	0.1553	−2.494	0.013
Deviation of 3 octants in wind direction (*D* = 3)	δ_3_	−1.166	0.1784	−6.534	<0.001
Deviation of 4 octants in wind direction (*D* = 4)	δ_4_	−0.729	0.1488	−4.901	<0.001

**Fig. 3:**
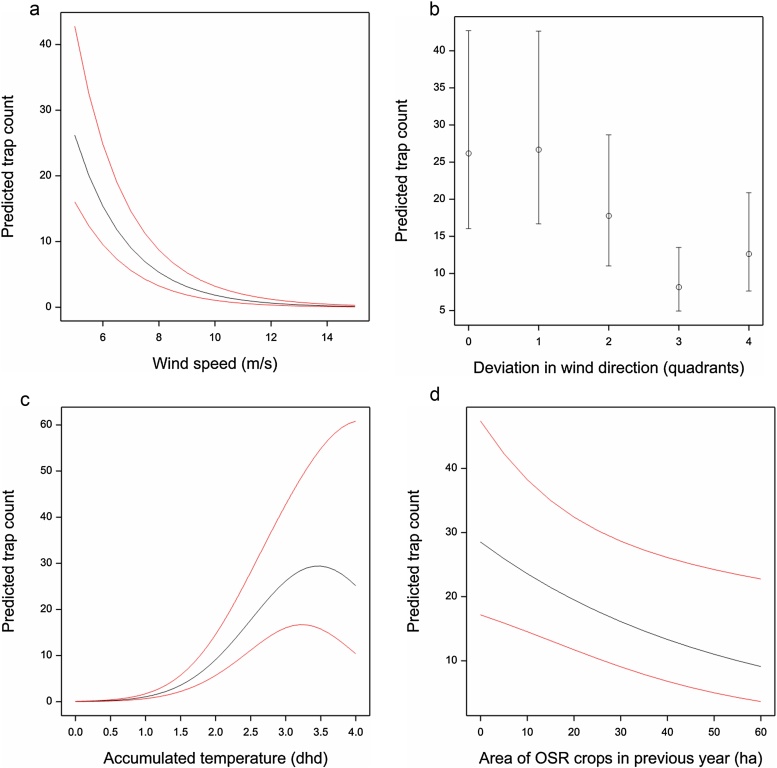
Predicted daily trap catch of pollen beetles with 95% confidence limits for (a) wind speed (m/s) with 3 dhd accumulated temperature, 4.5 ha oilseed rape (OSR) in landscape in previous year and trap aligned with wind direction, (b) the deviation between wind and trap direction in octants with wind speed of 5 m/s, 3 dhd accumulated temperature and 4.5 ha OSR in landscape in the previous year, (c) accumulated temperature (dhd) with windspeed of 5 m/s, 4.5 ha OSR in landscape in the previous year and trap aligned with wind direction, (d) the area of OSR crops within a 1000 m radius with windspeed of 5 m/s, 3 dhd accumulated temperature and trap aligned with wind direction.

There was a positive effect on daily total trap catches of being located on the Rothamsted estate, giving a predicted ratio of beetle numbers at Rothamsted relative to non-Rothamsted fields of 8.16, with 95% confidence interval (4.25, 15.68). Weather variables were again more important (i.e. with larger t-ratios) as explanatory variables than landscape features (Table 2). Wind speed (m/s) again showed a statistically significant negative linear relationship with log daily field catch (Table 2) shown in Fig. 3a with 95% confidence limits. Deviations of more than 1 octant between wind and trap direction were associated with lower trap catches (Table 2), although trap catches were slightly higher for 4 octants deviation (trap facing upwind) than for 3 octants deviation, which gave the lowest trap catches (shown in Fig. 3b). Accumulated temperature (dhd) showed a statistically significant positive linear relationship with log daily field catch, with a negative quadratic component (Table 2), shown in Fig. 3c. Relative field catches are expected to increase as accumulated temperature increases up to about 3.5 dhd (equivalent to a constant daytime temperature of 13.5 °C), at which point it plateaus and then decreases. Again rainfall was not significant and the only landscape variable showing statistical significance was a negative linear relationship on the log scale with area of OSR grown in the previous year (Fig. 3d).

## 4 Discussion

In our study, pollen beetle catches on sticky traps during beetle immigration into winter OSR crops were influenced by a combination of meteorological and landscape factors. At both field and single trap scales, meteorological influences, particularly wind speed and accumulated temperature, were dominant over landscape features as explanatory variables for trap catch, implying that the model could be used to predict periods of high beetle immigration risk, based on accumulated temperature and wind speed alone. Our results represent the most direct evidence to date that pollen beetles use upwind anemotaxis at a landscape scale during immigration into the crop. They also highlight that positioning of monitoring traps relative to field boundaries and wind direction is important in determining trap efficiency, and suggests that the area of OSR grown within the surrounding landscape in the previous season affects the abundance of beetles immigrating into OSR crops.

It is important to note that the models developed in this study are regression-based and are built on correlation between the explanatory variables and responses. Caution is therefore required when attributing causal effects to explanatory variables, as they may simply be representing the causal effect of a third variable that they are correlated with. At both the field and trap scales, larger numbers of pollen beetles were trapped at Rothamsted compared with other sites. These results were not unexpected due to the estate’s history of growing OSR crops without insecticide treatment, and both winter- and spring-sown OSR, but the magnitude of the differences (predicted catches at Rothamsted of c. 8–9 times those of other sites) was surprising. Over the last decade Rothamsted has, however, hosted many experiments investigating the trap cropping potential of turnip rape (*Brassica rapa* L.) for pollen beetle control. At the green bud stage, turnip rape is more attractive to pollen beetles than OSR, and the presence of a turnip rape border surrounding the crop can reduce within-crop beetle infestation to below spray threshold levels (Cook et al., 2004, Cook et al., 2006, Cook et al., 2007), but the early flowering of winter-sown turnip rape (if left untreated with insecticide) can lead to a large proportion of the pollen beetle larval population escaping parasitisation (Skellern et al. in prep), and a model developed by Vinatier et al. (2012) has suggested that turnip rape trap cropping could increase densities of the pest, especially if untreated. This, together with a very high proportion of gardens in the Rothamsted area relative to other sites could explain the larger populations of beetles found.

The insignificant influence of rainfall on trap catches, once accumulated temperature and wind speed had been accounted for, was unexpected as generally the activity of pollen beetles in the field appears reduced in wet conditions (S.M Cook and M. Skellern, personal observations) and precipitation of 1 mm was selected as a cut off value in previous models (Junk et al., 2016). However, there were relatively few days with >1 mm rain in our dataset (Fig. S5) and rainfall may tend to be associated with lower temperatures. In addition, other factors such as relative humidity and barometric pressure can affect insect flight activity (Fournier et al., 2005, Zhang et al., 2008, Tansey et al., 2010, Liu et al., 2011). It is possible, for example, that if elevated post-rainfall humidity conditions favour beetle migration (to our knowledge the influence of humidity on pollen beetle flight activity has not yet been characterised), this could have offset any negative influence of rainfall on trap catches.

The observation that pollen beetles were not found on traps until the maximum temperature rose above 10 °C is consistent with recent laboratory findings that the 10% flight temperature threshold for the pest lies within the range 10.9–12.5 °C (Ferguson et al., 2015), and with lower limits for field-observed flight of 10.2 °C (Laska and Kocourek, 1991) but higher than that predicted (8.0° C) by immigration models for beetles in Luxembourg (Junk et al., 2016). The quadratic effect of accumulated temperature indicated an increase in predicted beetle catch as accumulated temperature rose from 0 to 4.5 dhd for the field-scale model then predicted a decline. The trap-scale model showed a similar increase with accumulated temperature up to around 3.5 dhd, but then plateaued and predicted the decline at a slightly lower accumulated temperature. While pollen beetles show a sigmoidal temperature-response curve with 50% flight temperature threshold estimates of 15.5–16.2 °C (Ferguson et al., 2015), our predicted decrease in trap catch with higher temperatures for the trap-scale model should be regarded with caution; there were few observations above 4 dhds, so predictions for these higher temperatures may be unreliable, as indicated by the very wide confidence limits (Figs. 2 b and 3 c). It should also be borne in mind that during spring, higher temperatures are more likely to occur later in the season, towards the end of beetle immigration, when the available pool of beetles yet to migrate would be diminished. Hence, the observed catch may be lower than it would have been for the same environmental conditions earlier in the season.

Previous studies have indicated the tendency of pollen beetles to locate OSR crops by odour-driven upwind anemotaxis (Evans and Allen-Williams, 1994, Williams et al., 2007, Moser et al., 2009), but because we have used directional sticky traps placed at the field edge, we show this for the first time directly at the point of entry into an OSR crop − at a landscape scale. The largest numbers of beetles were caught over periods when traps were aligned with the downwind direction, suggestive of upwind anemotaxis. Accordingly, as the prevailing wind came from a WSW–SSW direction, those traps facing in a downwind direction (ENE) caught more beetles than upwind-facing (WSW) traps. These results indicate that for optimal trapping efficiency, pollen beetle monitoring traps should be placed on the downwind side of OSR crops, facing downwind relative to the prevailing wind direction. The tendency of beetles to fly upwind was clearly not an exclusive pattern, however, as beetles were still caught on traps facing other directions, but their numbers were lower, and tended to decrease as the trap faced away from the downwind direction. Interestingly, beetle numbers from traps where the deviation in trap–downwind direction was 4 octants (i.e. 180°) were higher than those from the cross-wind direction where the deviation was 3 octants (i.e. 135°); this observation is consistent with beetles sometimes travelling with the wind rather than flying upwind.

Previous studies that have investigated landscape effects of surrounding OSR area on pollen beetle abundance or herbivory in the same year have had variable results, but the majority, in agreement with the current study, have shown no influence (Thies and Tscharntke, 1999, Thies et al., 2003, Thies et al., 2008, Gladbach et al., 2011, Scheid et al., 2011, Rusch et al., 2013). Others have shown negative relationships, often attributed to dilution effects (Zaller et al., 2008a, Moser et al., 2009, Schneider et al., 2015), or positive relationships (Valantin-Morison et al., 2007). These discrepancies could arise from unknown differences among study regions (Rusch et al., 2013) or be attributable to sampling methodology differences, particularly in relation to scale, or to the temporal dynamics of the relationship between the beetles and their host crop. Indeed, Beduschi et al. (2015) showed that the effect of surrounding OSR area on beetle abundance changed with time, from being negative during peak flowering, to positive after flowering, probably reflecting dilution and crowding effects, respectively. In the current study, however, trapping took place mostly during the green–yellow bud stages, before flowering, when inward migrating beetle populations would have been concentrated around crop edges; any potential dilution effects of crop area on beetle abundance may not manifest until the later flowering stages, when the beetles are likely to be more dispersed.

Relatively few studies have considered the landscape-scale effects of OSR area in the previous season on the current season’s pollen beetle abundance. Schneider et al. (2015) showed that an inter-annual increase in landscape proportions of OSR resulted in beetle dilution effects, while Thies et al. (2008) observed no influence of this variable on pollen beetle herbivory. Beduschi et al. (2015) found that landscape proportions of OSR negatively influenced beetle abundance one or two years later, and that this effect was mediated through changes in parasitism. Although the present study shows landscape-scale effects of the previous season’s OSR area on the abundance of beetles immigrating into OSR crops, there were inconsistencies between the trap- and field scale models in the direction of these effects. While the field-scale model showed a positive influence of this variable, for the trap-scale model the relationship was negative. The landscape data used for the two models differed in that only landscape features facing the trap (in the same or neighbouring octants) were used in the trap-scale model whereas all surrounding landscape was used in the field-scale model, so we might not expect complete agreement. However, it is possible that these differences are the result of spurious correlation in one or both models as the distributions of previous season’s OSR crop areas and of the trap counts were both positively skewed, with many more occurring low than high values. If there was no relationship, there would likely be many fewer high trap counts for the few large areas simply because of the smaller number of observations. Exclusion of the top 10% of the distribution of previous season’s OSR crop areas from the analysis (analysis not shown) meant that the negative trap-scale relationship became non-significant, which supports this notion. However, excluding the top 10% of this distribution for the field-scale analysis increased the significance of the positive relationship, suggesting that, in contrast with the results of previous studies, this positive relationship may be real. Factors such as differences in parasitism rates and the extent to which regional scale landscape structure necessitates long-distance migration (Rusch et al., 2013) may explain these differences. For example, in landscapes that are reasonably balanced in terms of breeding and overwintering sites (limited migration necessary), it might be expected that, particularly where parasitism is low, beetle abundance may reflect surrounding OSR area in the previous year. By contrast, in landscapes that promote long distance migration because they are more compartmentalised at the regional scale (i.e. containing some areas with large open fields and other distinct areas with more complex landscapes), relationships between beetle abundance and the previous season’s OSR area are unlikely to be found, particularly at relatively fine (e.g. 1000 m) sampling scales.

Increasing proportions of woodland in the landscape are often associated with higher pollen beetle densities or damage (Valantin-Morison et al., 2007, Zaller et al., 2008b, Zaller et al., 2009, Rusch et al., 2012b, Rusch et al., 2013), probably due to their role as overwintering sites which later become the springtime source of emerging beetles. In the current study, however, the area of woodland in the surrounding landscape was not found to be an important determinant of trap catch, for either model. The reasons for this are unclear. As observed by Zaller et al. (2008b), the differing effects of landscape-related features among studies may relate to differences in methodological approach. The scale over which landscape features were mapped is important; if the scale was too large then the inclusion of irrelevant information (noise) may obscure relationships, or if the scale was too small, then relevant information would be missing. The 1000 m scale chosen is unlikely to have been inappropriate, however, as it was not dissimilar to those used by other studies that have shown effects of landscape features on pollen beetle abundance or damage (e.g. Thies et al., 2003, Zaller et al., 2008a, Moser et al., 2009, Beduschi et al., 2015, Schneider et al., 2015). The factors affecting beetle immigration may also be subtly different to those affecting beetle abundance on the crop, or crop damage, particularly when factors such as natural enemies and crop management are considered. Grassland habitats, which were not considered in the present study, can be important as pollen beetle overwintering sites (Rusch et al., 2012a) and as landscape-scale determinants of beetle abundance (Rusch et al., 2013), but Rusch et al. (2012a) also found that emerging beetles were more associated with local habitat characteristics such as low soil moisture and a thick litter layer than with habitat type *per se*. It is possible, particularly considering the wide geographical range of our study, that a range of habitats were providing conditions suitable for beetle overwintering; woodlands may have been important as overwintering sites in some regions, but in others different habitat types may have provided more suitable conditions, leading to a diminished effect of woodland area overall.

In conclusion, our study has shown that wind speed and accumulated temperature were more important than landscape variables in predicting the abundance of pollen beetles immigrating into OSR fields. The efficiency of pollen beetle monitoring traps could be optimised by placing them on the downwind side of a crop, facing downwind relative to the prevailing wind direction. The area of OSR crops grown in the surrounding landscape during the previous season was positively related to trap catch at the field scale and could potentially contribute to assessment of potential pest pressure for individual OSR crops. While proPlant.expert DSS already incorporates wind speed and accumulated temperature into its underlying model and has been shown to accurately predict periods of pollen beetle migration risk (Ferguson et al., 2016), optimal placement of monitoring traps could complement the DSS in terms of further reducing monitoring effort and costs. Inspection of the traps when the DSS has indicated a period of high migration risk would determine whether immigration into a specific crop has actually begun, and whether inspection of the crop is warranted, particularly as Ferguson et al. (2016), using the same data pool as the present study, found that at >91% of sites beetles were caught on monitoring traps before they were observed in the crop. The proPlant DSS is now freely available to UK growers and widespread DSS uptake by OSR growers is promising (Ferguson and Cook, 2014). This, in combination with the use of optimally-placed monitoring traps could help to reduce prophylactic insecticide applications, thus reducing impacts on non-target organisms and ameliorating the risk of further resistance development.
