# Supplementary material for: Meteorological and landscape influences on pollen beetle immigration into oilseed rape crops
Source: Agric Ecosyst Environ. 2017 Apr 1;241:150–9. doi: 10.1016/j.agee.2017.03.008 (PMC5485638; doi:10.1016/j.agee.2017.03.008)
Supplement: Supplementary file 1 [file mmc1.docx]

***S1. Simple exploratory model***

The exploratory model used trap catches as the response variable with a Poisson distribution and an offset to take account of the number of days that the trap was in the field. The model included terms for year, farm, field within year, sample date within field within year, trap direction (up- or down-wind), and trap direction within field within year. The residuals from this analysis and the mean squared residuals were inspected for indications of variance heterogeneity, in particular for any pattern of variance increasing as a quadratic function of the mean, which would be more consistent with a negative binomial distribution than with a Poisson distribution (ver Hoef & Boveng 2007).

The residuals from this analysis showed variance heterogeneity, with larger variance for larger fitted values, and the mean squared residuals also indicated a pattern of variance increasing as a quadratic function of the mean, more consistent with a negative binomial distribution. A negative binomial distribution with aggregation parameter k=5 gave acceptable residuals so this distribution was adopted for the model.

The exploratory model (see Table S3) showed strong evidence of variation in beetle counts between upwind, downwind and crosswind traps (F_3,501_=14.30, *P*>0.001), as well as differences from this overall pattern within individual fields. There was also strong evidence of variation between years, farms, and fields within farms. The model predicted average beetle counts per downwind and upwind trap of 98.7 (±22.50) and 60.2 (±12.66), respectively. Partitioning the sites into those at Rothamsted vs. others showed significantly higher counts at Rothamsted (F_1,501_=1312.5, *P*<0.001), with a predicted average per trap count of 139.4 beetles (±26.19) at Rothamsted as opposed to 50.5 (±8.68) from other sites. This difference was expected as, historically, many Rothamsted OSR fields (also including spring-sown crops) have been unsprayed to facilitate research. Although this difference affected beetle numbers, it was not expected to affect behaviour, thus an effect was added to all models to allow for higher numbers caught on traps at Rothamsted.

***S2. Details of fitted models***

*S2.1 Field-scale model*

The predicted total trap catch for the *i*th day in the *j*th field, γ_ij_(*w,R,a,p*), was modelled as

$$\log(\gamma_{ij})=\alpha+\beta_{1}R_{j}+\beta_{2}w_{ij}+\beta_{3}a_{ij}+\beta_{4}a_{ij}^{2}+\beta_{5}p_{j}+u_{j}$$

where *w_ij_* and *a_ij_* apply to the *i*th day in the *j*th field, and *R_j_*, *p_j_* and *u_j_* apply to all dates for the *j*th field. Specifically, *w_ij_* is the windspeed at 12:00 (m/s), *a_ij_* is the accumulated temperature (dhd), *R_j_* indicates whether the field is at Rothamsted (0/1), *p_j_* is the area under OSR in the previous year in the surrounding landscape (ha) and *u_j_* is the random effect for the *j*^th^ field. Estimates of the fixed effects are shown in Table 1. Catches over days within the sample period are accumulated to predict the total count, i.e. the expected catch for the *j*th field for a sample period running from day *d*_1_ to day *d*_m_ is

$$\mu_{j}(d_{1},d_{m})=\sum_{i=d_{1}}^{d_{m}} \gamma_{ij}(w_{ij},R_{j},a_{ij},p_{j}) .$$

These expected catches form the fitted values for the model.

*S2.2 Trap-scale model*

For the trap-scale model, the predicted catch for the *k*th trap on the *i*th day in the *j*th field, γ_ijk_(*w,R,a,p,D*), is a function of the explanatory variables modelled as

$$\log(\gamma_{ijk})=\alpha+\beta_{1}R_{j}+\beta_{2}w_{ij}+\beta_{3}a_{ij}+\beta_{4}a_{ij}^{2}+\beta_{5}p_{j}+\delta_{D_{ijk}}+u_{j}+v_{ij}+w_{jk}$$

where *w_ij_* and *a_ij_* apply to the *i*th day in the *j*th field, *R_j_* and *p_j_* apply to all dates for the *j*th field and *D_ijk_* applies to the *k*th trap on the *i*th day in the *j*th field. Specifically, *w_ij_* is the windspeed at 12:00, *a_ij_* is the accumulated temperature, *R_j_* indicates whether the field is at Rothamsted (0/1) and *p_j_* is the area under OSR in the previous year in the three trap-facing octants of the surrounding landscape. The function *D_ijk_* calculates the deviation of the downwind direction at 12:00 from the direction of the trap as number of octants (0–4), and a separate effect is fitted for each number of octants as δ*_l_*, *l* = 0,1,2,3,4. The model uses first-level-zero parameterization, so the estimated effect for a deviation of 0 octants is estimated as zero, and the effects for the other octants are relative to this value. For the random terms, *u_j_* is the effect of the *j*th field, *v_ij_* is the effect of the *i*th day in the *j*th field, and *w_jk_* is the effect of the *k*th trap in the *j*th field. Estimates of the fixed effects are shown in Table 2.

Catches over days within the sample period are accumulated to predict the total count, i.e. the expected catch for the *k*th trap in the jth field for a sample period running from day *d*_1_ to day *d*_m_ is

$$\mu_{jk}(d_{1},d_{m})=\sum_{i=d_{1}}^{d_{m}} \gamma_{ijk}(w_{jk},R_{j},a_{ij},p_{j},D_{ijk}) .$$

These expected catches form the fitted values for the model and can be compared to the observed trap catches.


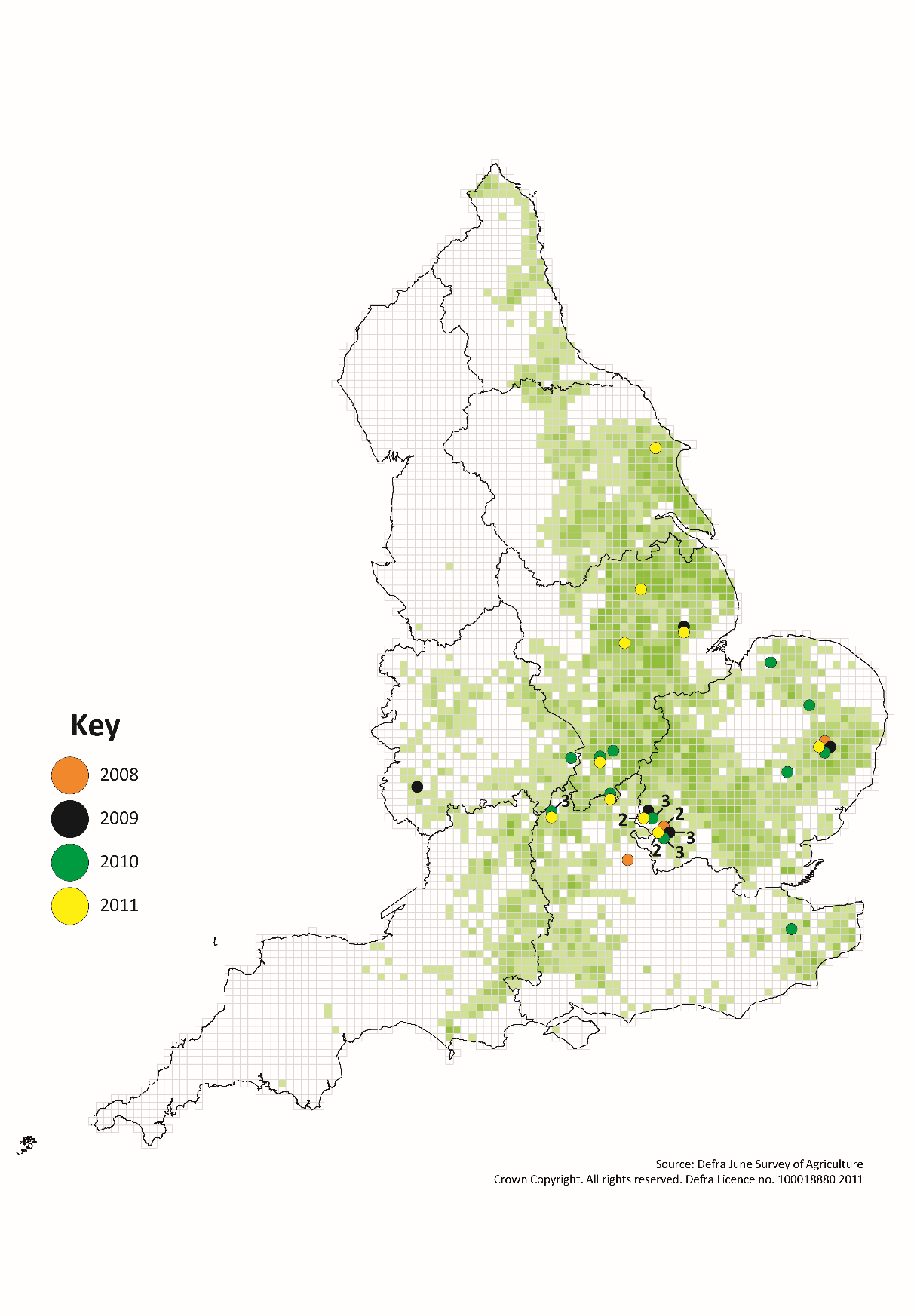


**Figure S1**. Map of England showing main areas of oilseed rape cropping (green shading) (Source: Defra June Survey of Agriculture) and location of sites used in each year of this study. Where more than one crop was used in per location in any one year, the number of sites is shown in numerals with an arrow relating to the corresponding year.


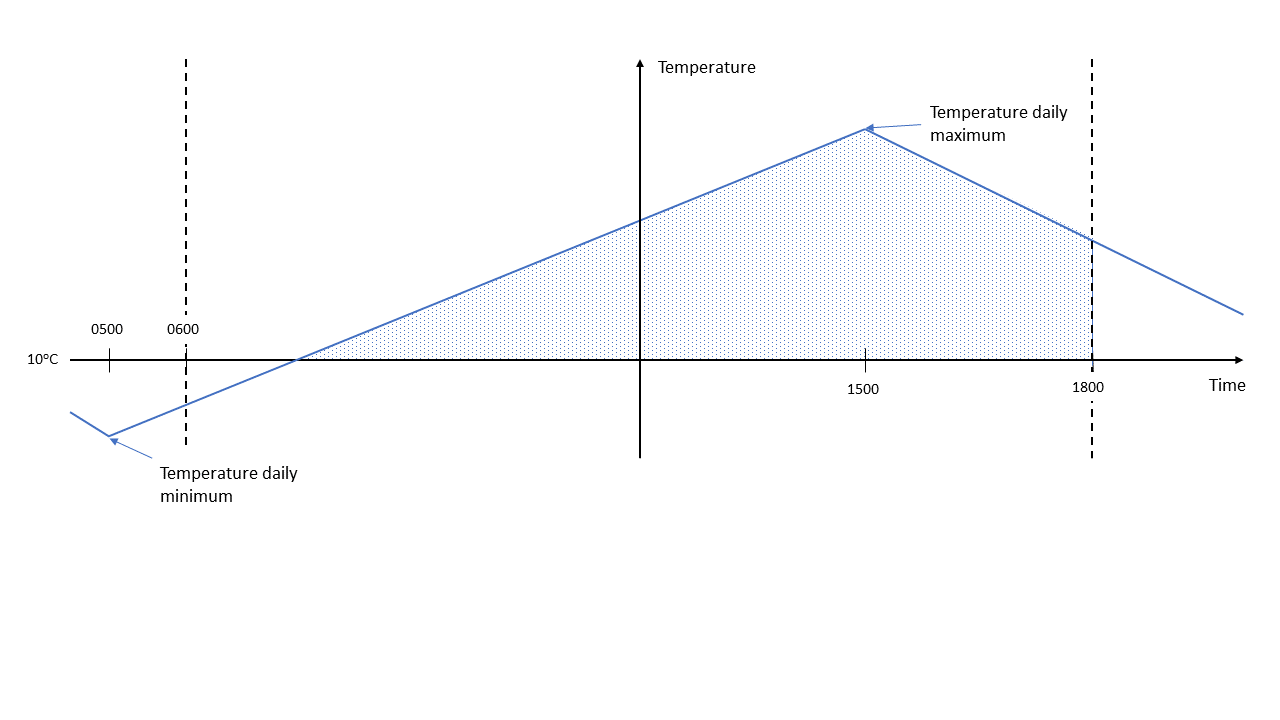


**Figure S2** Calculation of accumulated temperature. Temperature daily minimum is assigned at 0500 and maximum at 1500 and linear interpolation used at intermediate points (blue line). Daylight hours assigned as 0600 to 1800 (vertical dashed lines). Accumulated temperature (dhd, degree-half-days) was calculated as the integral above 10 ^o^C during the daylight period, shown as shaded area.


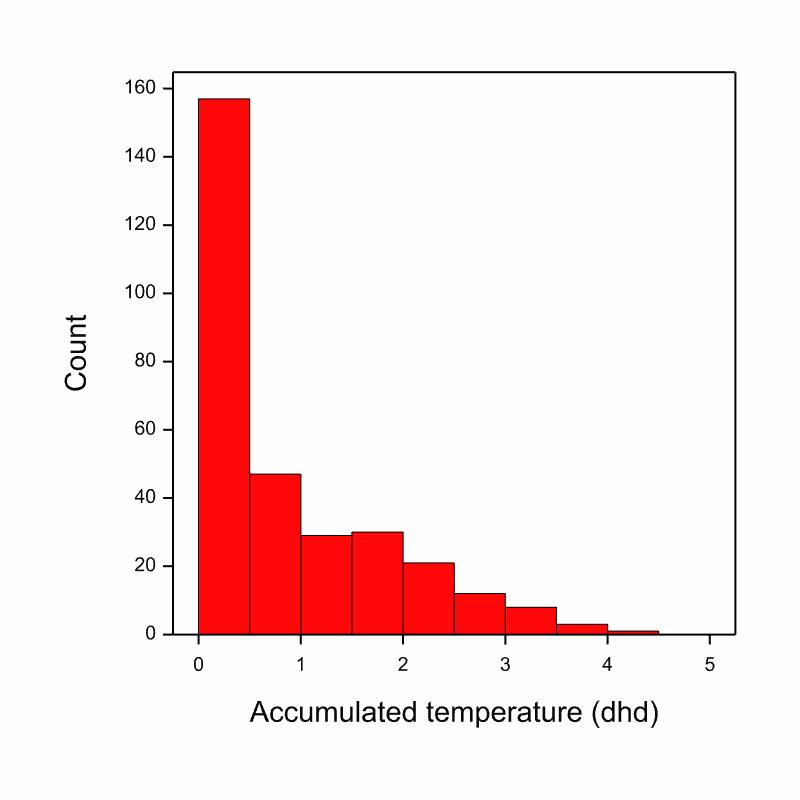


**Figure S3.** Distribution of accumulated temperatures (dhd) during sampling periods at field sites.


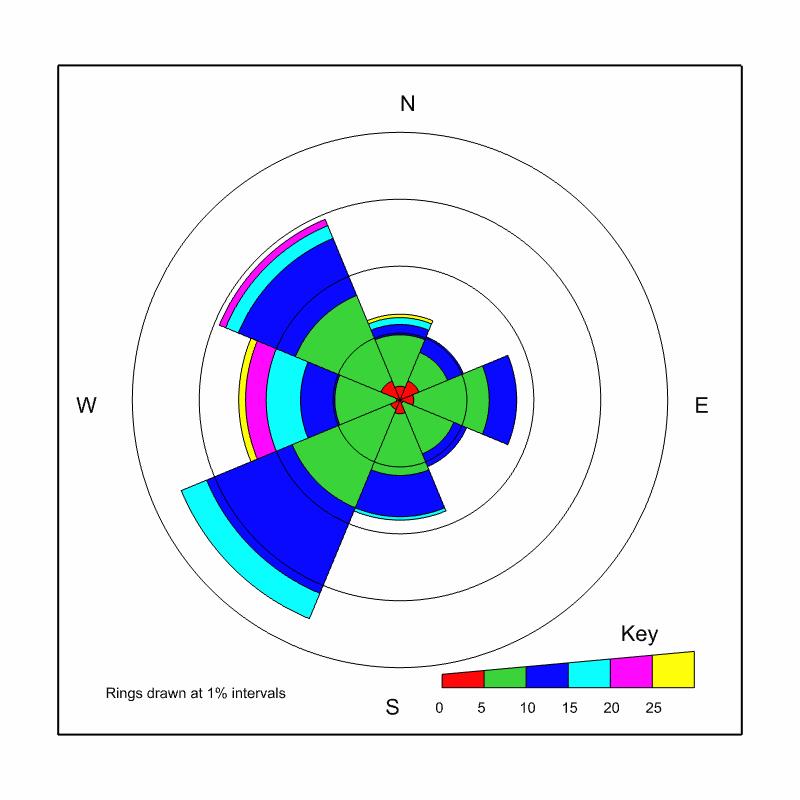


**Figure S4.** Distribution of wind speed by wind direction during sampling periods at field sites. The frequency of each direction determines the diameter in that direction. The colours (and key) relate to wind speed (m/s).


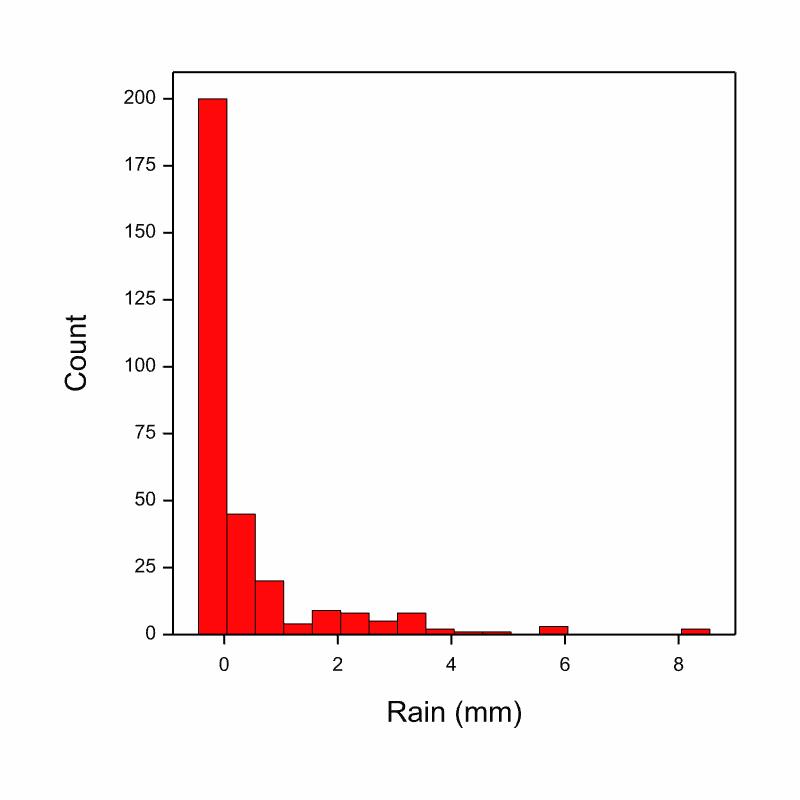


**Figure S5.** Distribution of daytime rainfall (mm) during sampling

| **Site name** | **Meteorological recording station** | **Approximate distance to meteorological recording station (km)** | **Number of samples used** | **Average growth stage of crop**  **(min-max)** | **Min number beetles**  **(upwind plus downwind traps only)** | **Peak number beetles**  **(upwind plus downwind traps only)** | **Julian date of peak** |
| --- | --- | --- | --- | --- | --- | --- | --- |
| GF_RRES_2008  (Hertfordshire) | Rothamsted | On farm | 5 | 55-59 | 0 | 238 | 94 |
| HB_2008  (Buckinghamshire) | SAWS Farnborough | 38.13 | 5 | 51-58.5 | 4 | 72 | 112 |
| Hoxne_2008  (Suffolk) | Wattisham | 29.71 | 4 | 53.5-57.5 | 0 | 49 | 91 |
| NZ_RRES_2008  (Hertfordshire) | Rothamsted | On farm | 9 | 55-62 | 0 | 185 | 112 |
| GK_RRES_2009  (Hertfordshire) | Rothamsted | On farm | 7 | 34-53.5 | 22 | 214 | 90 |
| Hoxne_2009  (Suffolk) | Wattisham | 29.30 | 12 | 18-59 | 0 | 91 | 99 |
| HP_Woburn_2009  (Bedfordshire) | Woburn | On farm | 14 | 13-56.6 | 0 | 111 | 110 |
| LH_RRES_2009  (Hertfordshire) | Rothamsted | On farm | 9 | 50-53 | 0 | 909 | 90 |
| LK_RRES_2009  (Hertfordshire) | Rothamsted | On farm | 12 | 15.5-54 | 0 | 677 | 99 |
| Rhea_2009  (Herefordshire) | SAWS Pershore | 12.94 | 8 | 37-53 | 0 | 92 | 89 |
| Stix_2009  (Lincolnshire) | Waddington | 18.40 | 9 | 31-57 | 0 | 281 | 89 |
| Allot_2010  (Warwickshire) | Church Lawford | 12.50 | 7 | 14-31 | 0 | 84 | 96 |
| BigI_2010  (Oxfordshire) | Brize Norton | 17.46 | 6 | 16-52 | 0 | 47 | 97 |
| Blis_2010  (Northamptonshire) | SAWS Bedford | 18.69 | 10 | 19-53 | 0 | 294 | 98 |
| BM_Woburn_2010  (Bedfordshire) | Woburn | On farm | 11 | 13.5-50 | 0 | 60 | 99 |
| Bozeat_2010  (Northamptonshire) | SAWS Bedford | 4.65 | 4 | 24.5-53 | 0 | 127 | 93 |
| Cowl_2010  (Suffolk) | Andrewsfield | 32.14 | 7 | 19.51.5 | 0 | 123 | 98 |
| DF_RRES_2010  (Hertfordshire) | Rothamsted | On farm | 11 | 15-53 | 0 | 784 | 97 |
| DH_RRES_2010  (Hertfordshire) | Rothamsted | On farm | 11 | 15-53 | 0 | 864 | 97 |
| EL_2010  (Kent) | Shoeburyness | 36.30 | 8 | 19-55 | 0 | 144 | 92 |
| GF_RRES_2010  (Hertfordshire) | Rothamsted | On farm | 11 | 15-53 | 0 | 643 | 97 |
| Hoxne_2010  (Suffolk) | Wattisham | 29.30 | 11 | 18-52 | 0 | 803 | 96 |
| HP_Woburn_2010  (Bedfordshire) | Woburn | On farm | 6 | 16-50.5 | 0 | 786 | 99 |
| MD_Woburn_2010  (Bedfordshire) | Woburn | On farm | 6 | 14-15.5 | 0 | 163 | 96 |
| Model_2010  (Oxfordshire) | Brize Norton | 20.01 | 11 | 18-55 | 0 | 532 | 100 |
| Morley_2010  (Norfolk) | Wattisham | 43.92 | 5 | 13.5-53 | 0 | 70 | 96 |
| Syd_2010  (Norfolk) | Marham | 26.84 | 5 | 20-53 | 0 | 810 | 96 |
| TG_2010  (Oxfordshire) | Brize Norton | 17.88 | 6 | 16-53 | 1 | 470 | 97 |
| Wicken_2010  (Northamptonshire) | SAWS Bedford | 21.35 | 11 | 16-55.5 | 0 | 615 | 99 |
| Blis_2011  (Northamptonshire) | SAWS Bedford | 18.69 | 5 | 19-31 | 0 | 210 | 80 |
| BM_Woburn_2011  (Bedfordshire) | Woburn | On farm | 5 | 19-45.5 | 0 | 613 | 80 |
| FW_2011  (Nottinghamshire) | Cottesmore | 30.60 | 2 | 50 | 63 | 213 | 80 |
| GF_RRES_2011  (Hertfordshire) | Rothamsted | On farm | 6 | 18-51.5 | 0 | 1284 | 80 |
| Harp_2011  (Lincolnshire) | Waddington | 25.92 | 3 | 30-43 | 0 | 226 | 78 |
| Hoxne_2011  (Suffolk) | Wattisham | 29.30 | 9 | 17-56 | 0 | 432 | 95 |
| MD_Woburn_2011  (Bedfordshire) | Woburn | On farm | 5 | 19-45.5 | 0 | 408 | 80 |
| Model_2011  (Oxfordshire) | Brize Norton | 20.01 | 7 | 32-51 | 0 | 360 | 80 |
| OS_RRES_2011  (Hertfordshire) | Rothamsted | On farm | 6 | 18-50.5 | 0 | 813 | 80 |
| Stix_2011  (Lincolnshire) | Waddington | 18.40 | 5 | 30-52 | 0 | 595 | 80 |
| Weav_2011  (Yorkshire) | Dishforth | 6.59 | 4 | 19-31 | 0 | 681 | 80 |
| Wicken_2011  (Northamptonshire) | SAWS Bedford | 20.10 | 9 | 14-55 | 0 | 206 | 96 |

**Table S1**. Details of meteorological recording stations, summary crop growth stage data and pollen beetle trapping data for the 41 sites included in the analyses. At each site, the Julian date and size of the peak beetle count was identified. All samples up to and including this date were used for analysis. Growth stages are averages of the values given at the first and last sample dates (min and max on each date, respectively).

| **Type** | **Variable** | **Minimum** | **Lower quartile** | **Median** | **Mean** | **Upper quartile** | **Maximum** |
| --- | --- | --- | --- | --- | --- | --- | --- |
| Area (ha) | Woodland | 3.6 | 18.8 | 33.4 | 36.6 | 42.6 | 134.8 |
|  | Gardens | 0.3 | 5.3 | 11.8 | 24.7 | 41.9 | 111.7 |
|  | OSR in current year | 2.8 | 11.0 | 33.7 | 41.4 | 63.8 | 131.3 |
|  | OSR in previous year | 0.0 | 4.4 | 12.1 | 21.1 | 38.7 | 99.0 |
| Length (km) | Treeline | 0.00 | 0.52 | 1.06 | 1.90 | 3.22 | 5.36 |
|  | Hedges | 2.03 | 6.16 | 9.82 | 10.09 | 13.58 | 25.06 |

**Table S2.** Summary of landscape data, mapped from within a 1000m-radius zone around each pollen beetle trap in fields containing oilseed rape (OSR) crops

| **Change** | **d.f.** | **Deviance** | **Mean deviance** | **Deviance ratio (F)** | **Approx. F prob.** |
| --- | --- | --- | --- | --- | --- |
| + Year | 3 | 832.234 | 277.411 | 199.16 | <0.001 |
| + Farm | 17 | 3476.291 | 204.488 | 146.80 | <0.001 |
| + Field | 20 | 824.450 | 41.222 | 29.59 | <0.001 |
| + Field.Sampledate | 276 | 18508.686 | 67.060 | 48.14 | <0.001 |
| + Trap | 3 | 59.740 | 19.913 | 14.30 | <0.001 |
| + Field.Trap | 68 | 511.570 | 7.523 | 5.40 | <0.001 |
| Residual | 501 | 697.859 | 1.393 |  |  |
| Total | 888 | 24910.831 | 28.053 |  |  |
|  |  |  |  |  |  |

**Table S3.** Sequential analysis of deviance (ANODEV) table from a simple exploratory model to ascertain sizes and sources of variability. Effects were fitted for year (Year), farm (Farm), field (Field), sample date within field (Field.Sampledate), trap direction (Trap), and trap direction within field (Field.Trap).
